# The impact of bilateral endoscopic inferior turbinoplasty with or without adenoidectomy on the quality of life of children: a retrospective case series study

**DOI:** 10.1186/s40463-019-0390-3

**Published:** 2019-12-02

**Authors:** Ahmed Mourad, Hussein Jaffal, Ismaeel El-Hakim, Hamdy El-Hakim

**Affiliations:** 1 Faculty of Medicine and Dentistry, University of Alberta, Edmonton, Alberta Canada; 2 Division of Otolaryngology – Head & Neck Surgery, Department of Surgery, The Stollery Children’s Hospital and University of Alberta Hospital, Edmonton, Alberta Canada; 3 Divisions of Otolaryngology – Head & Neck Surgery and Pediatric Surgery, Departments of Surgery and Pediatrics, The Stollery Children’s Hospital and University of Alberta Hospital, Edmonton, Alberta Canada

**Keywords:** Glasgow children’s benefit inventory, Chronic rhinitis, Quality of life, Pediatric, Inferior turbinoplasty

## Abstract

**Background:**

Inferior turbinoplasty (IT) and adenoidectomy (Ad) are frequently resorted to in children with chronic rhinitis (CR) refractory to medical therapy. The aim of this study is to document the long-term improvement in quality of life (QOL) in children with CR following endoscopic IT with or without Ad.

**Methods:**

A retrospective case series study was conducted. We searched a prospectively kept surgical database for children ≤18 years old who had CR who underwent endoscopic IT with or without Ad between 2009 and 2016 at a tertiary care children’s center. Patients with sinonasal pathologies other than CR, had craniofacial syndromes or dysmorphism and had other sinonasal procedures or trauma were excluded. Collected data included demographics, secondary diagnoses, duration of follow-up, and complications of procedures. The Glasgow Children’s Benefit Inventory (GCBI) was administered by phone to assess QOL improvement.

**Results:**

One hundred sixty-five eligible subjects were identified. Eighty-nine subjects met the inclusion criteria. Data was collected for the 60 subjects that were reached. Forty-two patients had IT only while 18 had IT and Ad. The mean age was 10.7 ± 2.7 years, with 31 males and 29 females. The median duration of follow-up (25th, 75th percentile) was 38.1 months (24.6, 55.8). The median GCBI score (25th, 75th percentile) was 22.9 (6.3, 39.6) revealing an overall positive benefit in all domains. There was only one complication.

**Conclusions:**

This study validates prior findings regarding improvement of QOL and safety of IT with or without Ad for children with CR and indicates it is maintained in the long term.

## Background

Chronic rhinitis (CR) is a longstanding inflammation of the nasal mucosa, which may develop in children secondary to allergic, vasomotor, infectious, immune-related, hormone-related, and/or medication-related etiologies. CR can manifest with daytime symptoms of nasal congestion/obstruction, rhinorrhea, mouth breathing, among others and is commonly associated with sleep-disordered breathing and snoring [1]. Upon assessment of a child with suspected CR, other etiologies of nasal obstruction or congestion must be looked for (for example adenoid hypertrophy, chronic rhinosinusitis with or without polyps, deviation of the nasal septum, and congenital anomalies etc.). If these etiologies are ruled out and the persistence of the clinical picture for more than 3 months is documented, then a diagnosis of CR can be established, and management planned accordingly. In patients who are suspected to have an allergic etiology or those who fail initial management, referral for assessment by an allergist is warranted. The mainstay of therapy consists of medical therapy, in the form of topical corticosteroids which may also be effective in patients with concomitant adenoid hypertrophy [1, 2]. Other medications are often added to enhance the response to therapy. Oral antihistamines and leukotriene antagonists are used especially when an allergic etiology is suspected. When medical therapy fails, adenoidectomy (Ad) is traditionally considered to be the next step especially when the adenoid tissue is significantly enlarged. However, a systematic review questioned the effectiveness of Ad in such a clinical scenario [3]. More recently, with the introduction of mucosa sparing technology and instruments of adequate caliber, surgery of the inferior turbinates found its way to the pediatric practice in the treatment of refractory CR. Several techniques have been described such as total or partial resection, mucosal or submucosal reduction using diathermy and coblation [4, 5]. However, microdebrider-assisted inferior turbinoplasty (IT) with the advantage of better mucosal preservation has been more widely accepted with documented efficiency and safety [6].

Besides the impact on nasal symptoms, CR can affect the quality of life (QOL) of children. Remarkably, a relatively modest number of studies have investigated this end point using validated measures. These instruments included Pediatric and Adolescent Rhinoconjunctivitis Quality of Life Questionnaire (PRQLQ) [4], Glasgow Children’s Benefit Inventory (GCBI) [5, 7], and the Sinus and Nasal QOL Survey (SN-5) [8]. Aside from some methodological misgivings and including different etiologies and techniques, these studies have mostly reported short term results.

We previously reported data on the efficacy and QOL improvement following IT with or without Ad in children with CR [7] which was in line with others. However, the mean follow-up was only 15.8 months. Therefore, the purpose of the current study is to assess the long-term impact in a different cohort of children.

## Methods

A retrospective, uncontrolled case study was conducted. A prospective surgical database was used for identifying eligible subjects among patients operated upon by one pediatric otolaryngologist, between July 2009 and November 2015 at a tertiary children’s referral center (Stollery Children’s Hospital, Edmonton, AB). The study was approved by the Health Ethics Research Board at the University of Alberta (Pro00065273).

Eligible subjects were patients 18 years of age or younger diagnosed with CR (allergic or non-allergic) and who had undergone bilateral endoscopic IT with or without Ad. CR was defined as symptoms of nasal congestion/obstruction lasting for at least 3 months and associated with nasal mucosal congestion. Consultation with an allergist was requested when an allergic etiology is suspected (itching, sneezing, circumstantial response to allergen exposure, seasonal symptoms, concomitant eczema, asthma, allergic conjunctivitis, family history of atopy) [1]. Surgical intervention was only offered after failure of a trial of maximal medical therapy which, in our practice, consisted of a compliant use of intranasal steroids for at least 3 months with or without an oral antihistamine or a leukotriene antagonist (where indicated). The included patients had to be followed up for a minimum of 3 months after surgery. Patients were excluded if they had other sinonasal pathologies such as significant septal deviation (more than 50% obstruction on nasal endoscopy) and nasal polyposis, as well as those with symptoms or signs suggestive of recurrent or chronic rhinosinusitis (headaches, facial pain, rhinorrhea, fever, requirement of antibiotics, and purulence on nasal endoscopy). Patients with craniofacial and nasal anomalies, syndromes, or trauma were excluded, as well as those who had immunodeficiency, central nervous system pathology, or other concomitant or prior sinonasal procedures.

We collected the following variables from the surgical database and hospital records (electronic or hard copies): age (in years), sex, secondary diagnoses, duration of follow-up (defined as the period from the surgery till the date the parents were contacted for this study in years), and complications of procedures (defined as these incidents requiring active management or re-admission to hospital). The main outcome measure was the Glasgow Children’s Benefit Inventory (GCBI) [9]. This is a validated QOL instrument which consists of 24 questions designed to retrospectively assess and measure benefit after an intervention in children. The overall scores reflect the change in the quality of life since the questions relate to the domains of emotion, physical health, learning, and vitality. Data from the GCBI was collected and quantified using a scale of − 100 to + 100, ranging from maximum harm to maximum benefit respectively, with a score of 0 indicating no change [9]. The parents of the included patients were contacted by telephone for up to three attempts to solicit recruitment. Once contact and verbal consent was achieved the parents were mailed a prepaid envelope to return their written consent. Additionally, we proposed an incentive of supporting a charity of their choice with one dollar to enhance the response rate. The GCBI questionnaire was administered directly by phone.

The decision to perform either IT alone or to combine it with Ad was based primarily on the size of adenoid tissue relative to the cross-sectional area of the posterior choanae as judged on nasal endoscopy in addition to the severity of CR [7]. The severity of CR was estimated based on a previously described grading system [10]. According to this system, CR is graded based on physical exam assessing for obstruction in the nasal cavities. CR is grade 1 if the is no obstruction (more than 50% compromise in patency) in either nasal cavity, grade 2 if the obstruction is unilateral, and grade 3 if the obstruction is bilateral. A standard technique was used for IT consisting of submucosal reduction using the microdebrider followed by out-fracturing of the bony concha [6]. Adenoidectomy was performed using a transoral monopolar diathermy technique [11].

Descriptive statistics were employed for summarizing the parameters of the patients. The Mann-Whitney signed-rank test was used when comparing medians. Statistical significance was set at *p* < 0.05. Box and whisker plots were used to graphically illustrate the results. SigmaStat version 4.0 was used for data analysis (Systat Software, San Jose, CA). The figures and tables were generated using Microsoft Excel and Microsoft Word (Office 365 ProPlus, Microsoft Corporation).

## Results

**Fig. 1:**
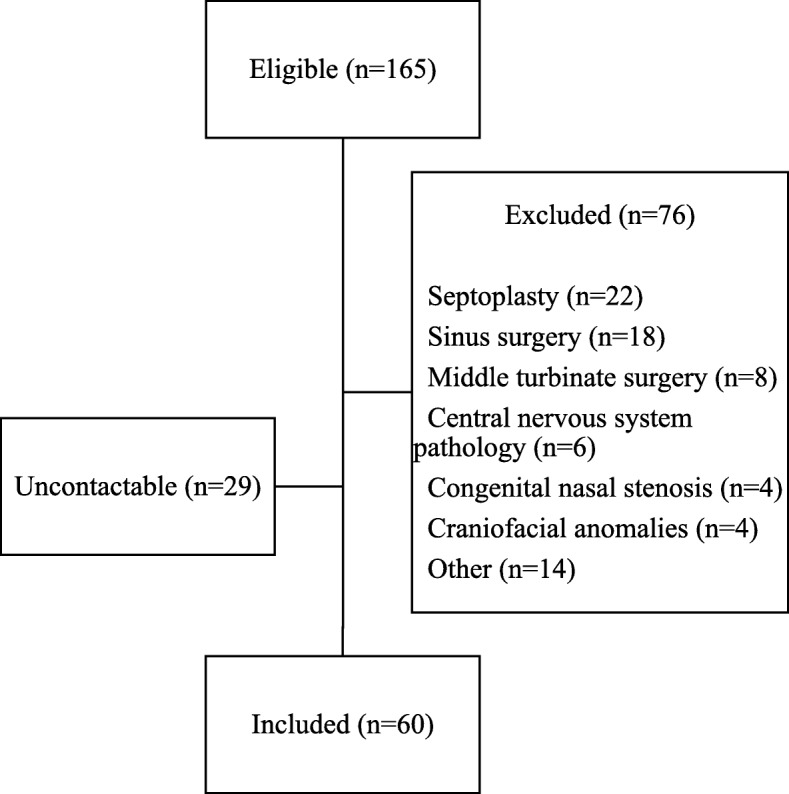
Flowchart illustrating the process of inclusion and the reasons for exclusion

One hundred sixty-five eligible subjects were identified from the surgical database. Seventy-six subjects were excluded based on the aforementioned criteria. The most common reason for exclusion was a concomitant septoplasty or sinus surgery (22 subjects and 18 subjects, respectively). Among the remaining 89, a total of 60 patients were contacted and their parents consented to participate. Twenty-nine families could not be contacted (Fig. 1).

We enrolled 31 males and 29 females in the study. Forty-two patients had IT only while 18 had IT and Ad. The mean age at the time of surgical intervention was 10.7 ± 2.7 years for all subjects, 10.7 ± 2.5 years for the IT only subgroup, and 10.9 ± 3.1 years for the IT and Ad subgroup. The median (25th, 75th percentile) duration of follow-up was 38.1 months (24.6, 55.8) (Table 1). Only one patient had a documented complication (toxic shock syndrome) that was managed with systemic antibiotics and intensive care admission (did not require assisted ventilation or inotropes) and developed no later sequalae. Only four patients had allergic rhinitis. Among the secondary diagnoses, the most common were sleep-disordered breathing (*n* = 13), obesity (*n* = 10), and asthma (*n* = 8). Three patients had concomitant chronic otitis media and two patients had malocclusion. The other diagnoses had one occurrence each (anxiety disorder, Asperger syndrome, history of prematurity, prolonged QT interval, sensorineural hearing loss).

**Table 1:** Age of patients and duration of follow-up

	N	Age (years)		Follow-up (months)	
		Mean ± SD	Range	Median (25th, 75th percentile)	Range
All Subjects	60	10.7 ± 2.7	4–15.4	38.1 (24.6, 55.8)	8.9–94.9
IT	42	10.7 ± 2.5	8.8–15.4	40.4 (24.6, 55.8)	8.9–94.9
IT + Ad	18	10.9 ± 3	4–14.7	35.4 (17.9, 40)	11.3–81.7

**Fig. 2:**
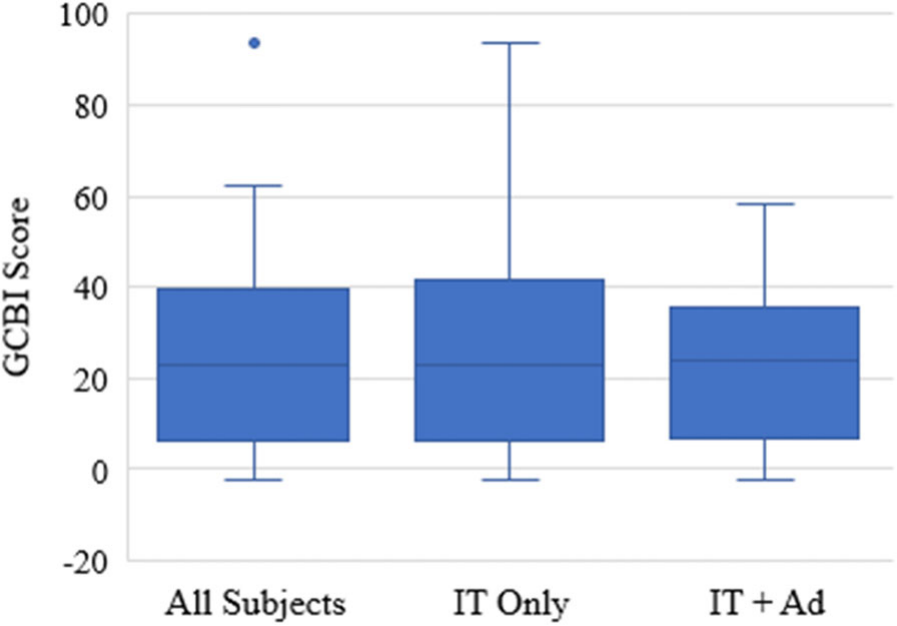
Box and whisker plot depicting the GCBI scores of all subjects and of each surgical subgroup. The boxes define the 25th and 75th percentiles, the whiskers define the 10th and 90th percentiles, and the dots represent the 5th and 95th percentiles

Analysis of the main outcome measure revealed a quality of life benefit in all domains with a median GCBI (25th, 75th percentile) of 22.9 (6.3, 39.6). The median scores for the surgical subgroups of IT only and IT + Ad were 22.9 (6.3, 41.7) and 24.0 (6.8, 35.4), respectively (Fig. 2) and there was no statistically significant difference between them (Mann-Whitney U Statistic = 369, *p* = 0.89). Two patients (3.3%) had a negative outcome (GCBI score = − 2), five (8.3%) reported no change (GCBI score = 0), and 53 (88.3%) had a positive GCBI score. None of the patients had revision surgery.

**Fig. 3:**
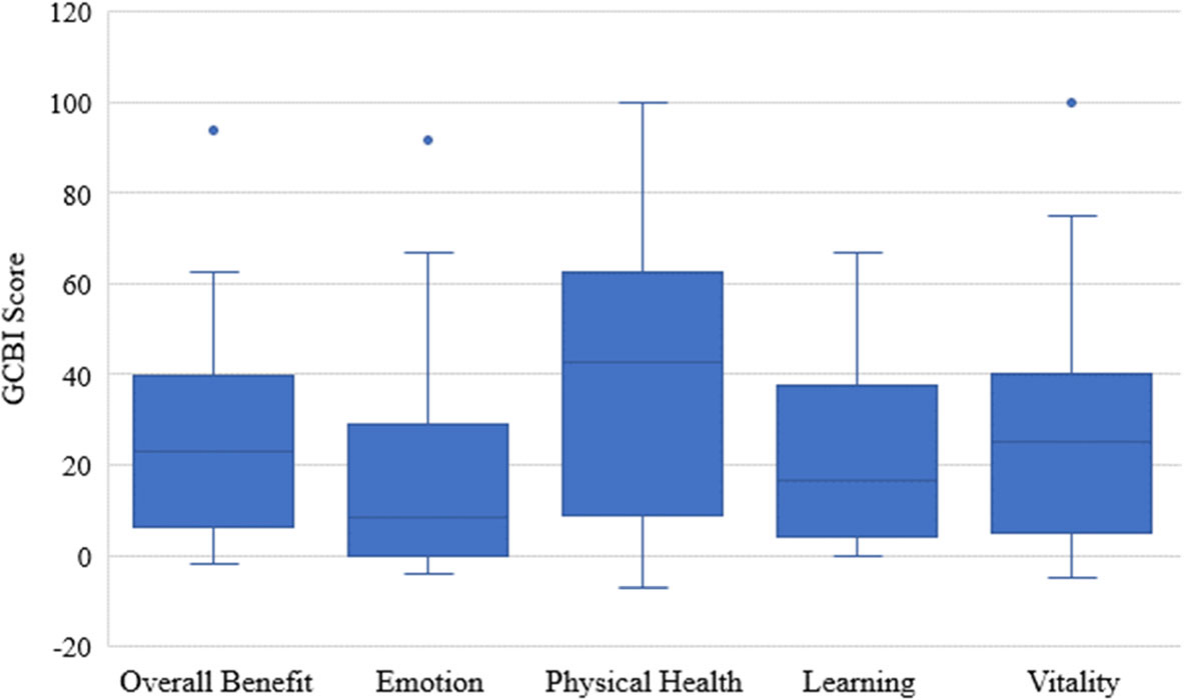
Box and whisker plot depicting the overall GCBI scores of all subjects and the scores for each of the four domains of QOL. The boxes define the 25th and 75th percentiles, the whiskers define the 10th and 90th percentiles, and the dots represent the 5th and 95th percentiles

Additionally, the individual median scores for each of the four QOL domains were calculated. Positive outcomes were noted for all domains, but the most prominent benefit was that of physical health with a median (25th, 75th percentile) of 42.9 (8.9, 62.5) (Fig. 3).

Finally, one of the questions in the GCBI questionnaire was the following: “Has your child’s operation affected how much medication he/she has needed to take?” The scores of this individual question were extracted and converted to a scale of – 100 to + 100. A median (25th, 75th percentile) score of 50 (0, 100) was obtained.

## Discussion

CR is a condition with significant implications on the health and well-being of affected children [1]. Surgical intervention is warranted in patients who do not respond favorably to medical therapy, with the mainstay being Ad and/or IT. The inclusion of IT in the initial surgical management is based on the questionable effectiveness of Ad [3].

Our results demonstrated an improvement in QOL after IT with or without Ad. This is in agreement with the results of prior studies that used validated QOL instruments following inferior turbinate surgery [4, 5, 7, 8] (Table 2). Simeon et al. studied prospectively the outcomes of coblation IT and showed improvement in nine subjects with allergic rhinitis (excluding those with a history of inferior turbinate surgery, adenoid hypertrophy, deviated nasal septum, and rhinosinusitis) using the Pediatric and Adolescent Rhinoconjunctivitis Quality of Life Questionnaire [4]. Aside from the small sample size, the follow-up period was relatively short (up to 6 months) and no information about the excluded subjects was provided. Montgomery et al. reported a larger sample (*n* = 47) of children who underwent submucosal diathermy of the inferior turbinates and again excluded those who had a simultaneous Ad [5]. No other exclusion criteria were adopted, and the presence of other confounding diagnoses was not discussed. In that case series 23 patients had a positive radio-allergosorbant test and the follow-up period was longer (ranging from 35 to 75 months). Their median overall GCBI score was 19.5 which is comparable to the current study. In another study, Manzi et al. reported an improvement in the Sinus and Nasal QOL Survey scores in all domains in 43 patients who underwent IT and out-fracturing [8]. They excluded those who had other nasal procedures including adenoidectomy. Similar to the other aforementioned studies, the presence of other diagnoses that might warrant exclusion of subjects is not clear, in fact the report documented the improvement of sinus infection. In this series, 14 out of 20 patients who underwent allergy testing had positive results (skin testing or IgE blood testing). The follow-up period reported was inconsistent, where 20 patients were only assessed at 1 to 2 months, and twenty-three for 1 to 2 years. The authors reported that the benefit was maintained in the latter group. In agreement with our results, they documented a decrease in the dependence on medical therapy.

**Table 2:** Previous studies on the change in QOL after inferior turbinate surgery using validated instruments

	Design	N	Intervention	Follow-up (months)	QOL Measures
Langille 2011 [7]	Retrospective	46	IT^a^ ± Ad	3–38	GCBI
Montgomery 2011 [5]	Retrospective	47	Submucosal diathermy	35–73	GCBI
Simeon 2010 [4]	Prospective	9	Coblation reduction	1–6	PRQLQ
Manzi 2017 [8]	Retrospective	43	IT^a^	1–24	SN-5

*GCBI* Glasgow Children’s Benefit Inventory. *PRQLQ* Pediatric and Adolescent Rhinoconjunctivitis Quality of Life Questionnaire. *SN-5* Sinus and Nasal QOL Survey

^a^Microdebrider-assisted submucosal reduction with outfracturing

The present study supports the previous work by Langille and El-Hakim who used the same methodology [7]. The main difference is the longer follow up duration (a median of 38.1 months compared to 15.8 months) and the larger sample size (60 vs 46). Long follow up is essential with such chronic conditions with a multitude of possible precipitants and exacerbating factors. Interestingly, the median GCBI score obtained after this longer follow up reflects a possible decrease in the benefit over time (22.9 vs 28.1). It is notable that both studies included hardly any patients with allergic rhinitis compared to the other studies (four in the current study and none in the study by Langille [7]). This is due to our standard approach to treat allergic rhinitis primarily with medical therapy which, with compliant use, can obviate the need for surgery.

Assessing both Ad and IT may seem out of the context of assessing the effectiveness of turbinate reduction surgery and confounds the impact of the two procedures. We feel this approach is warranted based on the following arguments. Since we accounted for the total cohort after exclusions it can be seen that nearly half of the patients had over 50% obstructive adenoids in addition to moderate enlargement (grade 2 according to the used scale). This means that a substantial proportion of the children presenting with CR may need to be addressed by two rather than one procedure. Also, although evidence is lacking on the effectiveness of Ad only, there is no body of work using a validated grading scale of CR that justifies a threshold for IT and argues for this procedure only in the presence of moderately enlarged adenoids. Lastly, we have been able to replicate our prior work that found similar benefit using IT only and those who had IT and Ad based on our criteria as evidenced by the lack of significant difference in the median GCBI scores between the surgical subgroups [7].

From the perspective of morbidity, only one patient developed toxic shock syndrome and our prior reported series documented no complications as well. This is not a complication that is specific to this particular surgery. The etiology was not clear although it may be secondary to packing with NasoPore®, a bioresorbable nasal dressing. We never encountered this complication in our practice despite our frequent use of nasal packing with IT. Further, Manzi et al. reported no adverse events of the technique, our study adds to the evidence on the safety of IT [8]. Similarly, there were no complications encountered with the other techniques (coblation and submucosal diathermy) [4, 5].

The GCBI is a validated scale which has been used to measure the outcomes of various interventions (surgical and non-surgical) in children. It allows insight into the specific domains of QOL: emotion, physical health, learning, and vitality.

One strength of the current study is the strict set of exclusion criteria. In order to best assess the benefit of IT in patients with CR, it is essential to exclude those with sinonasal pathologies other than CR and those who had concomitant septal or sinus procedures. Those factors, besides major comorbidities (e.g. craniofacial anomalies and syndromes) can be confounders when it comes to measuring the change in QOL.

The current study has several limitations. First, recall bias cannot be overlooked when a retrospective questionnaire is used especially when the follow up period is long, even though the instrument we used (the GCBI) is specifically designed to be used retrospectively. Second, the benefit cannot be extrapolated to patients with allergic rhinitis (4 patients out of 60). On the other hand, this indicates homogeneity in the study group and better generalizability to similar patients although we acknowledge that is lack of data on how many have undergone allergy testing. Third, 30 % of the eligible subjects was not contactable, even though several approaches were used to enhance the response rate. Finally, our measure of compliance with medical therapy was based only on direct questioning with no objective documentation.

## Conclusions

Overall, the data in the current study confirms that IT and IT and Ad are safe and their beneficial benefit in terms of QOL in children with CR refractory to medical treatment is maintained in the long term.

## Data Availability

The datasets used and/or analyzed during the current study are available from the corresponding author on reasonable request.

## Abbreviations

Ad: Adenoidectomy
CR: Chronic rhinitis
GCBI: Glasgow Children’s Benefit Inventory
IT: Inferior turbinoplasty
QOL: Quality of life
